# Enhancing academic resilience through mindfulness training: an experimental study with Chinese undergraduates and the mediating role of psychological flexibility

**DOI:** 10.3389/fpsyg.2025.1692295

**Published:** 2025-11-05

**Authors:** Ming Yuan, Zhe Hu

**Affiliations:** ^1^Department of General Education, Ziyang College of Dental Technology, Ziyang, China; ^2^Law School, Zhejiang University of Finance and Economics, Hangzhou, China

**Keywords:** mindfulness, self-compassion, psychological flexibility, academic resilience, university students, intervention, PLS-SEM, experimental design

## Abstract

**Introduction:**

This study investigates the impact of mindfulness on academic resilience among university students, with a particular focus on the mediating roles of self-compassion and psychological flexibility.

**Methods:**

Using a post-test-only control group experimental design, 200 undergraduate students from a Chinese university were randomly assigned to either an intervention group, which received a four-week mindfulness training program, or a control group. Data were collected after the intervention using validated self-report instruments and analyzed through Partial Least Squares Structural Equation Modeling (PLS-SEM).

**Results:**

The results revealed that mindfulness significantly enhanced both self-compassion and psychological flexibility, which in turn positively predicted academic resilience. Parallel mediation analysis confirmed that both self-compassion and psychological flexibility independently mediated the relationship between mindfulness and academic resilience, with stronger effects observed in the intervention group. Multi-group analysis further demonstrated significant differences in structural path strengths between the two groups, confirming the effectiveness of the mindfulness intervention.

**Discussion:**

These findings offer theoretical support for process-based models of resilience and emotion regulation and provide practical insights into scalable, low-cost interventions for enhancing student well-being in higher education. In addition, the study highlights culturally grounded evidence from a Chinese higher education context, addressing the relative scarcity of experimental investigations on mindfulness and resilience in non-Western settings. Limitations include the reliance on self-reported data, the use of a single-institution sample, and the short intervention period, which may constrain generalizability and long-term inference. Nevertheless, the findings support the relevance of mindfulness-based training as a culturally adaptable and resource-efficient approach to strengthening academic resilience in university populations.

## Introduction

The transition to university life poses considerable psychological demands on students, often marked by academic stress, emotional turbulence, and identity exploration. As educational environments grow increasingly competitive, the mental health and well-being of students have become central concerns for educators, psychologists, and policymakers alike (Hill et al., 2024). Conventional support services, though valuable, may not fully equip students with the internal tools required to navigate ongoing academic pressures. As such, there is growing interest in scalable psychological interventions that can foster resilience (Kakabaraei, 2024)—defined as the capacity to recover from adversity and maintain effective functioning.

Emerging from contemplative traditions and increasingly validated in psychological research, mindfulness offers a compelling framework for enhancing student resilience. Rather than promoting avoidance or emotional suppression, mindfulness encourages nonjudgmental awareness and acceptance of present-moment experiences (Si et al., 2024). Studies suggest that cultivating such awareness contributes not only to emotional stability but also to improved concentration, reduced anxiety, and enhanced academic performance (Erdemir et al., 2024; Zheng et al., 2024). However, understanding how mindfulness leads to these positive outcomes requires a deeper exploration of the mediating processes that may translate mindful awareness into resilient functioning.

While existing literature provides substantial evidence on the benefits of mindfulness in Western educational contexts (Allen et al., 2021; Bluth et al., 2023; Erdemir et al., 2024), relatively fewer studies have employed experimental designs to examine its mechanisms in non-Western settings, particularly within Asian higher education systems. In these contexts, academic pressure is often intensified by cultural expectations, competitive entrance examinations, and collectivist norms emphasizing achievement (Wu and Qin, 2025). Consequently, findings from Western samples may not generalize seamlessly to Asian student populations (Liu et al., 2024), where psychological processes and stress appraisals can differ substantially. Addressing this contextual gap is crucial for advancing both theoretical understanding and culturally sensitive intervention strategies.

Recent theoretical advances propose that the beneficial impact of mindfulness may operate through its influence on internal coping resources (Tenschert et al., 2024; Flook et al., 2025). One such mechanism involves the development of a kinder, more accepting attitude toward oneself during times of struggle. Instead of responding to academic setbacks with harsh self-criticism or avoidance, students who embody self-kindness are more likely to exhibit emotional balance and perseverance. This compassionate stance is not innate but can be cultivated, and research has increasingly recognized its role in fostering well-being across academic and clinical contexts. Still, empirical studies examining its mediating function within mindfulness-based frameworks remain limited, particularly in experimental settings.

In addition to compassionate self-relating, the capacity to adapt flexibly to internal experiences also plays a crucial role in managing stress. Rather than rigidly avoiding discomfort or reacting impulsively to negative emotions, psychologically flexible individuals can accept difficult thoughts while pursuing meaningful goals (Jeffords et al., 2020). This adaptability has been associated with a range of positive psychological outcomes, including reduced distress, greater life satisfaction, and improved problem-solving in educational settings (Alam and Mohanty, 2023). Given that mindfulness encourages acceptance and cognitive defusion, it is theoretically plausible that it enhances psychological flexibility, which in turn contributes to students’ ability to cope with academic adversity.

Despite these conceptual developments, the majority of studies linking mindfulness, self-compassion, psychological flexibility, and resilience are correlational or cross-sectional, making it difficult to infer causal mechanisms (Liu et al., 2024; Yosep et al., 2024). Moreover, interventions are rarely tested within rigorous experimental designs that allow for comparison between trained and untrained groups.

Equally important, most existing evidence originates from Western contexts, with limited attention to cultural nuances in Asia, where academic resilience is often shaped by distinct educational pressures and socio-emotional expectations. This lack of cultural grounding represents a significant research gap, particularly given calls for more context-sensitive psychological research (Zheng et al., 2024).

To address these limitations, the present study adopts a post-test-only control group experimental design (Wu and Qin, 2025) to examine how a structured mindfulness intervention influences academic resilience through two key intrapersonal mechanisms—self-compassion and psychological flexibility—among Chinese university students. By situating the investigation within a high-stakes academic culture and employing parallel mediation analysis, the study contributes both methodologically and contextually to the literature. This dual focus enhances its novelty compared to prior correlational or Western-centric studies, offering new insights into how mindfulness-based processes function within non-Western educational contexts.

By focusing on modifiable psychological pathways and using an experimental design, this study not only contributes empirical evidence to mindfulness research but also expands theoretical understanding of how inner resources can be cultivated to support well-being in higher education contexts. The findings aim to inform both scholarly discourse and practical interventions aimed at promoting mental resilience in student populations.

### Literature review

In response to the growing demands placed on university students, mindfulness has emerged as a valuable psychological resource that can be intentionally cultivated to enhance well-being and resilience. Mindfulness is commonly defined as a nonjudgmental awareness of present-moment experience, characterized by attentional regulation, openness, and acceptance (Sun et al., 2025). Practicing mindfulness helps individuals recognize and regulate automatic reactions to stressors, making it a promising approach for students navigating academic and emotional challenges. As a teachable and evidence-based construct, mindfulness has been associated with reduced anxiety, improved focus, and enhanced emotion regulation across student populations (Tenschert et al., 2024). The Mindfulness-to-Meaning Theory (Garland et al., 2015) further suggests that mindfulness initiates upward spirals of positive emotional processing, enabling individuals to derive adaptive meaning from adverse experiences.

One of the proposed psychological mechanisms linking mindfulness to improved functioning is self-compassion. Rather than harshly judging oneself during difficult times, self-compassion entails responding with kindness, mindfulness, and a sense of shared humanity (Neff, 2003, 2023). This construct comprises three dimensions: self-kindness versus self-judgment, common humanity versus isolation, and mindfulness versus over-identification. Introducing the “common humanity” dimension at the outset is particularly important, as it emphasizes recognizing one’s struggles as part of the broader human experience rather than signs of personal inadequacy—a perspective especially relevant in collectivist cultures where social comparison is prominent (Wu and Qin, 2025; Zheng et al., 2024). In academic contexts, self-compassion has been linked to decreased academic anxiety, greater emotional resilience, and improved motivation (Ewert et al., 2021). Since mindfulness fosters a non-reactive and accepting attitude toward internal experiences, it can enhance self-compassion by reducing self-criticism and increasing tolerance for personal failures (Per et al., 2022). As such, self-compassion is theorized to serve as an intrapersonal bridge between mindfulness and adaptive academic behaviors.

It is also important to clarify the conceptual distinction between mindfulness as a standalone psychological construct and the “mindfulness” component embedded within self-compassion. While both involve nonjudgmental awareness, the former reflects a broader attentional stance toward all experiences, whereas the latter pertains specifically to maintaining balanced awareness during moments of personal suffering (Neff, 2003, 2023). Confusing these two can lead to conceptual overlap in analyses (Grzybowski and Brinthaupt, 2022); thus, this study treats mindfulness and self-compassion as related but empirically distinct constructs, each measured separately and examined through parallel mediating pathways, consistent with recommendations to test discriminant validity between related psychological constructs (Bond et al., 2011; Brown et al., 2011). This distinction directly addresses concerns raised in prior scholarship about conflating these constructs in mediation models.

In addition to self-compassion, psychological flexibility is another central construct that may mediate the impact of mindfulness on academic resilience. Psychological flexibility refers to the ability to stay in contact with the present moment and adjust behavior in alignment with one’s values despite experiencing negative thoughts or emotions (Alam and Mohanty, 2023). According to Acceptance and Commitment Theory (ACT, Hayes et al., 2006), psychological flexibility underpins adaptive coping and mental health by reducing avoidance and increasing emotional openness (Bond et al., 2011). Mindfulness contributes to this process by promoting cognitive defusion, emotional acceptance, and value-driven action. In student settings, higher levels of psychological flexibility have been associated with lower levels of stress and greater persistence in the face of academic difficulty (Parker et al., 2024).

Finally, academic resilience refers to a student’s capacity to effectively manage academic demands, recover from setbacks, and maintain motivation and performance over time (Rudd et al., 2021). Unlike general resilience, which captures broad life functioning, academic resilience is context-specific and reflects goal-directed perseverance within the educational domain. Empirical studies have identified mindfulness, self-compassion, and psychological flexibility as key contributors to academic resilience, suggesting that students who embody these traits are better equipped to manage academic pressure and emotional disruption (Abdollahi and Isanejad, 2024; Salsabila and Widyasari, 2021). However, most existing work in this area is cross-sectional, limiting our understanding of how these constructs interact in real-time learning environments.

However, despite the promising theoretical models linking these constructs, much of the existing literature remains descriptive and lacks critical synthesis. For example, while several studies highlight positive associations between mindfulness and resilience (Erdemir et al., 2024; Tenschert et al., 2024), others point to contextual variations and the influence of cultural factors that may shape these relationships (Wu and Qin, 2025; Zheng et al., 2024). Similarly, the mediating roles of self-compassion and psychological flexibility have often been tested in isolation rather than in parallel (Jennings et al., 2023; Parker et al., 2024), limiting the ability to discern their relative contributions. By integrating these constructs within a parallel mediation framework and testing them through a controlled experiment in a non-Western educational setting, the present study seeks to address these gaps and provide a more nuanced understanding of how internal psychological resources interact to foster resilience.

**Figure 1:**
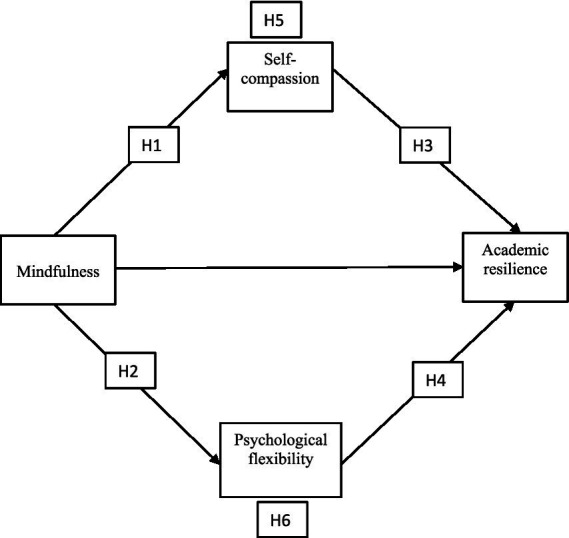
Proposed theoretical model.

Given the theoretical and empirical basis for these constructs, the present study seeks to examine a parallel mediation model in which mindfulness influences academic resilience through the simultaneous effects of self-compassion and psychological flexibility. By applying an experimental design within a university context, this research aims to clarify the causal mechanisms underpinning student resilience and offer evidence-based pathways for psychological intervention (Figure 1).

### Hypotheses

Given the increasing psychological burden on university students, cultivating internal emotional resources has become essential for sustaining both well-being and academic engagement. Accordingly, the study posits that mindfulness may exert its most transformative impact when viewed as a precursor to other self-related adaptive processes—particularly self-compassion. The Mindfulness-to-Meaning Theory (Garland et al., 2015) supports this conceptual pathway by arguing that mindfulness initiates cognitive reappraisal processes that allow individuals to reinterpret suffering in a more compassionate and constructive light. Furthermore, mindfulness fosters decentering—the ability to observe one’s thoughts and emotions without becoming entangled in them—which directly counters self-criticism and enables a gentler self-attitude (Guo, 2024). Empirical evidence reinforces this connection. For instance, Per et al. (2022) demonstrated that participants in an eight-week Mindful Self-Compassion program showed significant increases in self-compassion compared to a control group. Similarly, Grzybowski and Brinthaupt (2022) found that trait mindfulness significantly predicted levels of self-compassion among undergraduate students. Moreover, a longitudinal study by Lee et al. (2021) highlighted that increases in mindfulness over time were accompanied by parallel improvements in self-compassion, suggesting a directional and developmental relationship. These findings are echoed in diverse populations, from healthcare workers (Orosa-Duarte et al., 2021) to adolescents (Bluth et al., 2023), confirming the generalizability of this link. Taken together, these theoretical and empirical contributions provide a strong basis to infer that mindfulness directly contributes to the cultivation of self-compassion. Accordingly, it is hypothesized that:

*H1:* Mindfulness will have a significant positive effect on self-compassion among university students.

In addition, mindfulness contributes meaningfully to the development of psychological flexibility by promoting an open and accepting awareness of internal experiences (Cherry et al., 2021), thereby reducing experiential avoidance (Bond et al., 2011)—a key barrier to psychological adjustment. Theoretically, this relationship is well-articulated within the framework of Acceptance and Commitment Therapy (Hayes et al., 2006), which positions mindfulness as a core process that undermines experiential avoidance and promotes acceptance. Furthermore, mindfulness facilitates cognitive defusion (Fang et al., 2022), helping individuals observe their thoughts without identifying with them, thereby enabling greater adaptability and resilience during stressful academic situations. In this perspective, Martínez-Rubio et al. (2021) found that students with higher levels of mindfulness reported significantly greater psychological flexibility and lower levels of distress. In addition, a randomized controlled trial by Zarvijani et al. (2021) demonstrated that mindfulness-based interventions led to substantial improvements in psychological flexibility among college students. More recently, Lachaud and Louis (2024) confirmed that brief mindfulness training could increase tolerance for uncertainty and reduce cognitive rigidity, both of which are vital for flexible coping. Moreover, Mitsea et al. (2022) highlighted that mindfulness fosters metacognitive awareness, enabling students to disengage from unhelpful thought loops and choose adaptive responses. Taken together, these findings provide robust theoretical and empirical support for the inference that mindfulness enhances psychological flexibility. Therefore, it is hypothesized that:

*H2:* Mindfulness will have a significant positive effect on psychological flexibility among university students.

The study further anticipates that students frequently encounter academic setbacks such as poor grades, performance anxiety, or critical feedback, all of which can undermine their motivation and well-being. One such critical resource is self-compassion, which helps students reframe academic difficulties through a lens of understanding rather than harsh self-criticism (Wakelin et al., 2022). According to O’Hare and Gemelli (2023), students who treat themselves with kindness when facing academic stressors are less likely to ruminate on setbacks and more likely to re-engage with learning tasks effectively. In a study by Ewert et al. (2021), self-compassion was found to predict adaptive coping and lower levels of anxiety and depression among college students. Furthermore, Munroe et al. (2022) demonstrated that self-compassion attenuates the fear of failure, allowing students to persist after academic disappointments. Jennings et al. (2023) added that self-compassion promotes self-regulated behaviors such as goal setting and self-monitoring, both of which are essential components of academic resilience. In another study by Volkaert et al. (2022), adolescents with higher self-compassion reported greater perceived competence and stress tolerance during academic transitions. Taken together, these findings suggest that self-compassion fosters emotional balance and self-acceptance, which in turn cultivate the psychological stability needed to persist through academic adversity. Therefore, it is reasonable to infer that:

*H3:* Self-compassion will have a positive effect on academic resilience among university students.

In addition to its role in emotional regulation, psychological flexibility serves as a foundational mechanism that enables students to persist in the face of academic adversity. According to Zarvijani et al. (2021), this adaptive capacity is especially relevant in academic environments, where uncertainty, setbacks, and pressure are routine. Theoretically, Acceptance and Commitment Theory (Hayes et al., 2006) positions psychological flexibility as central to human flourishing, suggesting that those high in flexibility are better equipped to adaptively manage distress and maintain functioning during challenging circumstances. Furthermore, the resilience literature increasingly conceptualizes resilience not only as a trait but as a dynamic process shaped by psychological strategies such as openness, acceptance, and committed action (Alam and Mohanty, 2023). For example, Szarko et al. (2022) found that higher levels of psychological flexibility were strongly associated with greater academic persistence and lower academic burnout. Similarly, Cherry et al. (2021) reported that students with greater flexibility demonstrated enhanced coping efficacy and were more likely to recover from academic setbacks. Moreover, Erdemir et al. (2024) showed that flexibility predicted academic adjustment and emotional resilience in university populations across both Western and Eastern contexts. These findings collectively highlight that psychological flexibility enables students to confront academic challenges with greater adaptability and perseverance. Accordingly, it is hypothesized that:

*H4:* Psychological flexibility will have a positive effect on academic resilience among university students.

Building on the established direct relationships between mindfulness and self-compassion (H1), as well as between self-compassion and academic resilience (H3), it is theoretically plausible to infer a mediating pathway in which self-compassion serves as the mechanism through which mindfulness enhances academic resilience. Similarly, the direct link between mindfulness and psychological flexibility (H2), alongside the influence of psychological flexibility on academic resilience (H4), provides a conceptual basis for another mediating route. These propositions are consistent with the Mindfulness-to-Meaning Theory (Garland et al., 2015), which posits that mindfulness fosters adaptive emotion regulation processes—such as self-kindness and openness to inner experience—that in turn enhance one’s capacity to recover from stress. From a process-based view, this model suggests that mindfulness operates not in isolation but by cultivating intrapersonal resources that mediate its positive outcomes. Prior research supports these pathways: for instance, Grzybowski and Brinthaupt (2022) found that increases in self-compassion explained the effects of mindfulness interventions on emotional resilience, while Parker et al. (2024) emphasized psychological flexibility as a core pathway linking mindfulness to adaptive functioning. Therefore, based on both theoretical integration and empirical precedent, it is hypothesized that:

*H5:* Self-compassion mediates the relationship between mindfulness and academic resilience among university students.

*H6:* Psychological flexibility mediates the relationship between mindfulness and academic resilience among university students.

## Methodology

This study adopted a post-test-only control group experimental design, which is widely recognized for establishing causal relationships while minimizing threats to internal validity such as testing effects (Campbell and Stanley, 2015; Creswell and Creswell, 2017). Participants were randomly assigned to either an intervention group, which received a structured mindfulness-based program, or a control group that did not receive any psychological intervention during the study period. This design allowed for an unbiased comparison of outcomes across groups after the intervention, enabling a direct assessment of the impact of mindfulness training on self-compassion, psychological flexibility, and academic resilience.

The post-test-only design was selected because it effectively reduces sensitization or practice effects that can occur in pre–post designs, particularly when using psychological self-report instruments in short interventions (Campbell and Stanley, 2015). This approach is especially appropriate when random assignment is used, as it allows strong causal inference without repeated measurement bias.

A total of 200 university students (*n* = 100 per group) from a major Chinese institution participated in the study. Recruitment was conducted through campus-wide announcements, and informed consent was obtained prior to participation. The required sample size was determined through an *a priori* power analysis using G*Power 3.1, targeting a medium effect size (*f*^2^ = 0.15), *α* = 0.05, and power = 0.80 for a model with three predictors. The analysis indicated that a minimum of 77 participants per group was required; our final sample of 100 per group therefore provided sufficient power for detecting medium effects in the structural model.

The mindfulness intervention lasted for 4 weeks and involved weekly guided sessions focused on cultivating present-moment awareness, nonjudgmental acceptance, and emotional adaptability. Each session lasted approximately 60 min and followed a structured sequence: (1) introduction to mindfulness concepts and psychoeducation (week 1), (2) formal practices including breath awareness and body scan meditations (week 2), (3) application to academic stress scenarios and self-compassion exercises (week 3), and (4) integration and reflection on practice for academic resilience (week 4). The sessions were facilitated by trained instructors with prior certification in mindfulness-based approaches. Intervention fidelity was maintained through standardized manuals, session checklists, and weekly instructor supervision meetings to ensure protocol adherence.

At the end of the intervention period, both groups completed the same set of standardized instruments. The specific scales used to assess mindfulness, self-compassion, psychological flexibility, and academic resilience—including item counts, response formats, and psychometric properties—are fully detailed in the Measures section below. The Mindful Attention Awareness Scale – Adolescent version (MAAS-A; Brown et al., 2011) was selected for this study because of its established validity among university populations and its sensitivity to short-term interventions, making it appropriate for the four-week program. All measures demonstrated high internal reliability in the present context.

Data analysis was conducted using SmartPLS 4.0, which provides advanced PLS-SEM capabilities suitable for models involving mediators and moderators, particularly in small to moderate samples (Sarstedt et al., 2021). In addition to standard reliability, validity, and path analyses, model fit was assessed using the standardized root mean square residual (SRMR) and normed fit index (NFI), providing complementary information about global model adequacy. The main analysis included group-wise assessments of reliability, collinearity, model fit, and structural path coefficients. Multi-group analysis (MGA) was also performed to compare the structural relationships across the intervention and control groups and to examine whether the mindfulness-based intervention produced statistically significant differences in psychological outcomes.

### Demographics

Table 1 presents the demographic profiles of participants in both the control and intervention groups, each comprising 100 university students. The gender distribution was nearly balanced across both groups, with males representing 48% of the control group and 50% of the intervention group. The majority of participants were between the ages of 18 and 23, with a slightly higher proportion of 21–23-year-olds in the intervention group (50%) compared to the control group (45%). In terms of academic year, both groups included students from all four years, though the intervention group had a marginally higher representation in the second and third years. Regarding academic discipline, participants were fairly evenly distributed across social sciences, natural sciences, engineering, and arts and humanities, indicating a diverse and comparable sample in both groups, suitable for controlled comparisons in the subsequent experimental analysis.

**Table 1:** Demographic profiles of participants.

Variable	Control group (*n* = 100)	Intervention group (*n* = 100)
Gender		
Male	48 (48%)	50 (50%)
Female	52 (52%)	50 (50%)
Age group		
18–20	35 (35%)	32 (32%)
21–23	45 (45%)	50 (50%)
24+	20 (20%)	18 (18%)
Year of study		
First year	25 (25%)	20 (20%)
Second year	30 (30%)	35 (35%)
Third year	25 (25%)	30 (30%)
Fourth year	20 (20%)	15 (15%)
Academic discipline		
Social sciences	30 (30%)	28 (28%)
Natural sciences	25 (25%)	27 (27%)
Engineering	30 (30%)	28 (28%)
Arts and humanities	15 (15%)	17 (17%)

### Measures

To assess the core constructs of this study, standardized and validated self-report measures were employed. Mindfulness was measured using the 14-item *Mindful Attention Awareness Scale–Adolescent version (MAAS-A)* developed by Brown et al. (2011), which assesses present-moment attention and awareness. Self-compassion was evaluated using the 26-item *Self-Compassion Scale (SCS)* developed by Neff (2003) and adapted into Turkish by Akın et al. (2007), measuring components such as self-kindness, common humanity, and mindfulness. Psychological flexibility, a key mediator in this study, was assessed using the 7-item *Acceptance and Action Questionnaire-II (AAQ-II)* developed by Bond et al. (2011), which captures individuals’ ability to accept internal experiences while engaging in values-consistent behavior. Resilience was measured using the 14-item *Resilience Scale (RS-14)*, revised by Wagnild (2011) from the original RS-25, to assess internal strengths and adaptability in the face of adversity. All items across scales were rated on five-point Likert scales ranging from 1 (strongly disagree) to 5 (strongly agree). The internal consistency for each scale was acceptable in both the control and intervention groups, with Cronbach’s alpha values exceeding 0.70, confirming the reliability of the instruments for use in the current research context.

## Results

Table 2 summarizes the reliability and validity statistics for mindfulness, self-compassion, psychological flexibility, and academic resilience across the control group, intervention group, and the complete sample. All constructs exhibited high internal consistency, with Cronbach’s alpha (*α*) ranging from 0.802 to 0.874 and composite reliability (CR) between 0.860 and 0.915, exceeding the recommended threshold of 0.70. Average variance extracted (AVE) values for all constructs were above 0.50 (0.567–0.671), indicating satisfactory convergent validity.

**Table 2:** Reliability and validity assessment (psychometric properties of constructs).

Construct	Control group			Intervention group			Complete		
	Α	CR	AVE	α	CR	AVE	*α*	CR	AVE
Mindfulness	0.821	0.878	0.591	0.832	0.887	0.602	0.827	0.882	0.597
Self-compassion	0.845	0.894	0.624	0.857	0.906	0.637	0.851	0.900	0.631
Psychological flexibility	0.802	0.860	0.567	0.818	0.873	0.573	0.810	0.867	0.570
Academic resilience	0.861	0.902	0.658	0.874	0.915	0.671	0.867	0.909	0.665

Academic resilience demonstrated the strongest reliability (α = 0.861–0.874), followed by self-compassion (α = 0.845–0.857), mindfulness (α = 0.821–0.832), and psychological flexibility (α = 0.802–0.818). These results confirm that the measurement instruments performed consistently across both groups, providing a solid psychometric foundation for subsequent structural analyses.

Table 3 displays the variance inflation factor (VIF) values for all predictor constructs across the control group, intervention group, and the complete sample. All VIF values fell well below the conventional threshold of 5.0, ranging from approximately 1.298 to 2.231. This indicates that no multicollinearity issues were present among the predictors—mindfulness, self-compassion, psychological flexibility, and academic resilience—ensuring stable and unbiased parameter estimates in the structural model.

**Table 3:** Variation inflation factor (collinearity assessment).

Predictor	Control group				Intervention group				Complete			
	1	2	3	4	1	2	3	4	1	2	3	4
1. Mindfulness		2.134	1.456			2.231	1.372			2.182	1.412	
2. Self-compassion			1.312	1.765			1.298	1.834			1.305	1.802
3. Psychological flexibility		1.312		1.842		1.298		1.751		1.305		1.793
4. Academic resilience		1.765	1.842			1.834	1.751			1.802	1.793	

Table 4 presents the structural path coefficients for the control group, intervention group, and the complete sample. All hypothesized structural relationships were statistically significant (*p* < 0.001) across groups, with stronger effects observed in the intervention group.

**Table 4:** Path analysis.

Path	Control group				Intervention group				Complete			
	B	T	P	Result	B	T	P	Result	B	T	P	Result
Mindfulness → self-compassion	0.398	5.987	0.000	Accept	0.502	7.821	0.000	Accept	0.451	9.101	0.000	Accept
Mindfulness → psychological flexibility	0.365	5.611	0.000	Accept	0.435	7.098	0.000	Accept	0.400	8.217	0.000	Accept
Self-compassion → academic resilience	0.285	4.512	0.000	Accept	0.368	5.978	0.000	Accept	0.328	7.541	0.000	Accept
Psychological flexibility → academic resilience	0.312	4.101	0.000	Accept	0.374	5.598	0.000	Accept	0.343	6.811	0.000	Accept
Mindfulness → self-compassion → academic resilience	0.113	3.489	0.000	Accept	0.174	5.087	0.000	Accept	0.146	5.890	0.000	Accept
Mindfulness → psychological flexibility → academic resilience	0.398	5.987	0.000	Accept	0.502	7.821	0.000	Accept	0.451	9.101	0.000	Accept
Mindfulness → academic resilience (Total)	0.365	5.611	0.000	Accept	0.435	7.098	0.000	Accept	0.400	8.217	0.000	Accept

For the intervention group, mindfulness had significant positive effects on self-compassion (*B* = 0.502, *T* = 7.821, *p* < 0.001) and psychological flexibility (*B* = 0.435, *T* = 7.098, *p* < 0.001). Both mediators, in turn, significantly predicted academic resilience (self-compassion: *B* = 0.368, *T* = 5.978, *p* < 0.001; psychological flexibility: *B* = 0.374, *T* = 5.598, *p* < 0.001). The indirect effect of mindfulness through self-compassion was 0.174 (*T* = 5.087, *p* < 0.001), and the indirect effect through psychological flexibility was also statistically significant. The total effect of mindfulness on academic resilience remained significant (*B* = 0.435, *T* = 7.098, *p* < 0.001).

For the control group, similar directional relationships were found, but path coefficients were comparatively smaller (e.g., mindfulness → academic resilience: *B* = 0.365, *T* = 5.611, *p* < 0.001; self-compassion → academic resilience: *B* = 0.285, *T* = 4.512, *p* < 0.001). The pooled model confirmed that all hypothesized paths remained significant (*p* < 0.001), demonstrating the robustness of the structural framework.

Overall, these results support the hypothesized mediation model, indicating that mindfulness exerts both direct and indirect effects on academic resilience through self-compassion and psychological flexibility, with stronger pathways evident under the intervention condition.

Table 5 reports the explanatory power (*R*^2^), predictive relevance (*Q*^2^), and effect sizes (*f*^2^) for the endogenous constructs—self-compassion, psychological flexibility, and academic resilience—across the control group, intervention group, and the complete sample. *R*^2^ values were moderate to substantial, indicating that the model explained a meaningful proportion of variance in all endogenous variables. For self-compassion, *R*^2^ increased from 0.388 in the control group to 0.521 in the intervention group; for psychological flexibility, *R*^2^ rose from 0.375 to 0.480; and for academic resilience, it improved from 0.561 to 0.628. In addition, *Q*^2^ values were positive across all variables (0.210–0.374), confirming the model’s predictive relevance. Similarly, *f*^2^ values fell in the small to medium range (0.119–0.335), indicating that the predictors contributed meaningfully to the variance explained in each construct. Overall, the explanatory and predictive power of the model was stronger under the intervention condition, reflecting the effectiveness of the mindfulness program in shaping the key psychological outcomes.

**Table 5:** Regression analysis.

Endogenous variables	Control group			Intervention group			Complete		
	*R^2^*	*Q^2^*	*f^2^*	*R^2^*	*Q^2^*	*f^2^*	*R^2^*	*Q^2^*	*f^2^*
Self-compassion	0.388	0.219	0.132	0.521	0.289	0.182	0.456	0.251	0.154
Psychological flexibility	0.375	0.210	0.119	0.480	0.273	0.168	0.432	0.243	0.142
Academic resilience	0.561	0.325	0.283	0.628	0.374	0.335	0.597	0.349	0.309

Table 6 presents the MGA results comparing structural path differences between the intervention and control groups. Significant differences emerged for all key direct and indirect relationships, indicating that the mindfulness intervention strengthened the structural pathways leading to academic resilience.

**Table 6:** Multigroup analysis (MGA).

Relationships	Difference (Intervention – control)	*p*-value
Mindfulness → self-compassion	0.104	0.001
Mindfulness → psychological flexibility	0.070	0.014
Self-compassion → academic resilience	0.083	0.003
Psychological flexibility → academic resilience	0.062	0.011
Mindfulness → self-compassion → academic resilience	0.061	0.005
Mindfulness → psychological flexibility → academic resilience	0.049	0.016
Mindfulness → academic resilience (Total)	0.071	0.000

For the direct paths, the intervention group showed stronger effects for mindfulness → self-compassion (*Δ* = 0.104, *p* = 0.001), mindfulness → psychological flexibility (Δ = 0.070, *p* = 0.014), self-compassion → academic resilience (Δ = 0.083, *p* = 0.003), and psychological flexibility → academic resilience (Δ = 0.062, *p* = 0.011). Both indirect paths were also significantly stronger under the intervention condition: mindfulness → self-compassion → academic resilience (Δ = 0.061, *p* = 0.005) and mindfulness → psychological flexibility → academic resilience (Δ = 0.049, *p* = 0.016). Finally, the total effect of mindfulness on academic resilience was significantly larger in the intervention group (Δ = 0.071, *p* < 0.001). These findings collectively demonstrate that the intervention amplified both direct and mediated effects, reinforcing its overall effectiveness.

## Discussion

The discussion addresses a critical gap in experimental mindfulness research within non-Western higher education contexts, offering both conceptual and contextual insights into the mechanisms that underpin student resilience. This study set out to examine how mindfulness influences academic resilience among university students through the parallel mediating roles of self-compassion and psychological flexibility, using a controlled experimental design. The findings provide clear support for the hypothesized model, revealing that mindfulness exerted both direct and indirect effects on academic resilience, with stronger pathways evident among students who received mindfulness training. By integrating parallel mediation analysis with multigroup comparison, the study offers a rigorous account of how structured mindfulness interventions shape students’ psychological capacities and adaptive academic outcomes.

The findings indicate that mindfulness significantly enhanced self-compassion and psychological flexibility, which in turn predicted higher academic resilience. These results resonate with the Mindfulness-to-Meaning Theory (Garland et al., 2015), which posits that mindfulness initiates upward spirals of cognitive and emotional transformation, allowing individuals to reinterpret stressors adaptively. In particular, the robust mindfulness–self-compassion pathway aligns with Neff’s (2003, 2023) conceptualization of self-compassion as a regulatory resource that counteracts harsh self-judgment and cultivates balanced emotional responses. Previous studies have documented similar associations (e.g., Cosantino, 2021; Kramer et al., 2023), but by demonstrating this relationship in a randomized experimental design, the present study provides stronger causal evidence than prior correlational work.

The parallel pathway through psychological flexibility also yielded significant results, supporting Acceptance and Commitment Theory (Hayes et al., 2006). Mindfulness training enabled students to adopt a more open and adaptive stance toward their internal experiences, consistent with findings by Ewert et al. (2021) and Flook et al. (2025). This underscores the role of mindfulness not merely as a state of awareness but as a catalyst for adaptive cognitive and emotional processing that supports academic resilience.

The mediation results are theoretically important because they clarify *how* mindfulness contributes to resilience. Rather than treating self-compassion and psychological flexibility as simple correlates, the findings position these constructs as active mechanisms through which mindfulness exerts its effects. This aligns with calls in the literature for a shift from trait-based to process-based models of resilience, emphasizing malleable psychological pathways over static attributes.

The multigroup analysis results offer further theoretical insight. All key structural relationships were significantly stronger in the intervention group, indicating that structured mindfulness training amplifies both direct and mediated effects. This pattern suggests that intervention fosters more efficient and consolidated psychological processes, rather than merely enhancing dispositional mindfulness. In other words, the intervention did not simply increase students’ mindfulness scores but strengthened the functional links between mindfulness, compassion, flexibility, and resilience. This finding advances theoretical discussions by illustrating that mindfulness-based mechanisms can be actively trained and optimized, rather than being fixed personal traits.

Cultural context adds another layer of significance. Much of the empirical work on mindfulness and resilience has emerged from Western contexts that emphasize individual self-regulation. By contrast, this study took place within Chinese higher education, a collectivist environment characterized by intense academic competition and strong social comparison pressures. The prominent role of self-compassion—particularly its “common humanity” dimension—may hold special importance in collectivist cultures, where feelings of isolation and face concerns are prominent (Wu and Qin, 2025; Zheng et al., 2024). By documenting how mindfulness training strengthens these culturally relevant psychological pathways, the study provides context-sensitive evidence that enriches cross-cultural mindfulness scholarship.

In sum, the Discussion highlights that structured mindfulness training not only enhances students’ psychological resources but also strengthens the underlying mechanisms that enable resilience. By engaging critically with theoretical models and situating the findings in their cultural context, this study advances both conceptual understanding and empirical clarity regarding the pathways through which mindfulness operates in academic settings.

### Theoretical implications

This study makes several important theoretical contributions to the literature on mindfulness, self-compassion, psychological flexibility, and academic resilience. First, by employing an experimental design, the study provides causal evidence for the role of mindfulness in enhancing self-compassion and psychological flexibility, thereby strengthening the theoretical foundations of both the Mindfulness-to-Meaning Theory (Garland et al., 2015) and Self-Compassion Theory (Neff, 2003, 2023). Whereas much of the existing research in this domain has relied on cross-sectional or correlational data, this study demonstrates that structured mindfulness training actively cultivates these psychological mechanisms, moving beyond trait-based interpretations toward process-oriented models of resilience.

Second, the simultaneous testing of self-compassion and psychological flexibility as parallel mediators represents a novel contribution. Previous studies have typically examined these mechanisms in isolation, limiting theoretical understanding of how multiple pathways may operate concurrently. By integrating both mediators into a unified model and validating this structure empirically, the study enriches theoretical explanations of how mindfulness exerts its effects. This parallel mediation approach provides greater explanatory precision, offering a more comprehensive account of the cognitive-affective processes that underlie resilience.

Third, the multigroup analysis offers a significant theoretical insight by demonstrating that mindfulness-based mechanisms can be actively strengthened through intervention. The significantly stronger direct and indirect effects in the intervention group highlight that mindfulness operates not only as a dispositional factor but also as a trainable set of processes. This finding advances current theoretical debates by emphasizing the plasticity of self-compassion and psychological flexibility, positioning them as modifiable mediators rather than fixed traits.

Finally, by conducting the study in a Chinese higher education context, this research adds a valuable cross-cultural dimension to existing theories of mindfulness and resilience. Much theoretical work in this area has been developed in Western contexts; demonstrating the applicability of these mechanisms within a collectivist cultural setting contributes to the cultural validity and generalizability of current frameworks. The salience of self-compassion’s “common humanity” component in this context also points to culturally specific nuances that existing theories should more fully account for.

Taken together, these contributions deepen theoretical understanding by linking mindfulness with trainable psychological mechanisms, validating parallel mediation structures, and broadening the cultural scope of existing frameworks.

### Practical implications

The findings of this study offer several concrete and actionable implications for educational institutions, mental health practitioners, and policymakers seeking to enhance student well-being and academic resilience. First, the demonstrated effectiveness of a short, four-week mindfulness intervention highlights the feasibility of integrating structured mindfulness programs into university settings without significant resource burdens. This is particularly relevant for institutions with large student populations and limited counseling capacity, where scalable, cost-effective approaches are urgently needed.

Second, the evidence that mindfulness enhances both self-compassion and psychological flexibility suggests that interventions should go beyond teaching basic mindfulness exercises to explicitly cultivate these two psychological capacities. This could involve embedding compassion-based reflective practices and acceptance-based coping strategies within mindfulness curricula to strengthen their impact on resilience. For example, brief workshops, semester-long elective courses, or digital self-paced modules could be designed to progressively build these skills in a structured manner.

Third, the finding that intervention strengthened both direct and mediated effects underscores the importance of experiential, repeated mindfulness practice rather than one-off exposure. Educational leaders should therefore prioritize sustained implementation—for instance, through regular mindfulness sessions embedded within academic schedules, student orientation programs, or peer-facilitated practice groups. This shift from passive awareness-raising to active skill-building could lead to more meaningful and lasting outcomes.

Fourth, the cultural context of the study offers valuable practical insights for universities in collectivist societies, such as China. Because self-compassion and psychological flexibility can be tailored to address cultural values like common humanity and social harmony, program designers can adapt content to resonate with local cultural norms while retaining psychological effectiveness. This cultural sensitivity can increase student engagement and intervention relevance.

Finally, these findings provide a clear rationale for universities and educational policymakers to institutionalize mindfulness-based approaches as part of broader student support frameworks. Interventions targeting self-compassion and psychological flexibility can be strategically deployed for early prevention of burnout, reduction of academic stress, and promotion of adaptive coping among at-risk students. By integrating mindfulness-based initiatives at multiple levels—curriculum, counseling, and policy—universities can cultivate more resilient academic communities.

### Limitations

Despite its contributions, this study has several limitations that should be acknowledged and addressed in future research. First, the use of a post-test-only control group experimental design, while effective in minimizing testing effects, limits the ability to examine within-person change over time. Future studies could employ longitudinal or pre–post designs with multiple follow-ups to assess the sustainability and temporal dynamics of intervention effects on self-compassion, psychological flexibility, and resilience.

Second, all variables were measured through self-report instruments, which raises the possibility of common method bias and social desirability effects. Although validated measures were used and psychometric properties were strong, future research should incorporate behavioral indicators, peer reports, or physiological measures (e.g., heart rate variability, attentional tasks) to triangulate findings and strengthen causal inference.

Third, the study was conducted in a single Chinese university, which may limit the generalizability of the findings to other cultural or educational contexts. Future work could expand to multiple institutions and diverse cultural settings, enabling cross-cultural comparisons and increasing external validity.

Fourth, the intervention lasted only 4 weeks, which, while effective, does not reveal the long-term maintenance of the observed effects. Future studies should examine the durability of these outcomes through extended interventions and follow-up periods, ideally tracking whether gains in self-compassion, psychological flexibility, and resilience persist over semesters or academic years.

Finally, the study did not include potential moderating variables such as baseline stress, prior mindfulness experience, or personality traits, which may shape individual responsiveness to mindfulness interventions. Subsequent research could integrate these moderators into the model, providing a more nuanced understanding of which students benefit most and under what conditions.

Addressing these limitations will help build a more comprehensive and generalizable evidence base on how mindfulness-based mechanisms operate across time, contexts, and individual differences.

## Conclusion

In conclusion, this study provides robust evidence that mindfulness enhances academic resilience both directly and through the parallel mediating mechanisms of self-compassion and psychological flexibility, with these effects significantly amplified by structured mindfulness training. By combining an experimental design with parallel mediation and multigroup analyses, the study moves beyond correlational findings to offer causal and process-based insights into how mindfulness operates within academic contexts. Situated within Chinese higher education, the findings extend existing theoretical frameworks into a collectivist cultural setting, highlighting culturally relevant psychological processes such as common humanity. Practically, the results underscore the value of integrating short, scalable mindfulness programs into university structures to strengthen students’ adaptive capacities. Taken together, this research advances theoretical understanding, broadens cultural perspectives, and informs educational practice, offering a meaningful contribution to the literature on mindfulness and student resilience.

## Data Availability

The raw data supporting the conclusions of this article will be made available by the authors, without undue reservation.
